# Effect of levamisole, Vitamin E, and selenium against aflatoxicosis in broilers chicken

**DOI:** 10.14202/vetworld.2018.248-253

**Published:** 2018-02-24

**Authors:** Amjed H. Ulaiwi

**Affiliations:** Department of Pathology and Poultry Diseases, College of Veterinary Medicine, University of Baghdad, Iraq

**Keywords:** aflatoxin, aspartate transaminase, alanine transaminase, broiler, levamisole, Vitamin E and selenium

## Abstract

**Aim:**

The experiment was conducted to determine of levamisole (0.2 ml/kg-BW), Vitamin E (80 mg)+selenium (1.6 mg), and aflatoxin (B1) (positive control) compared with group without aflatoxin (negative control) on some liver enzymes (aspartate transaminase [AST] and alanine transaminase [ALT]), as well as to study the histopathological changes.

**Materials and Methods:**

The experiment included (200) 1-day-old broilers Ross 308 (Turkey source) mixed sexes. They were divided into four equal groups (50 chicks each group). The experimental period was extended to 35 days.

**Results:**

The results revealed that the levels of liver enzymes (ALT and AST) of all groups at 35 days were significantly (p<0.05) higher than the negative control. Furthermore, the result of histopathological changes in thymus and Harderian gland in different ages of group Vitamin E+selenium showed a reduction in the depletion of the cortex as well as lessening of congestion and hemorrhage and necrosis also decreasing in inflammatory cells in the thymus and Harderian gland.

**Conclusion:**

The study confirmed the protective effect of Vitamin E and levamisole by reducing harmful impacts of aflatoxin through their antioxidant effect as they improved the liver enzymes and histopathological changes due to the toxin.

## Introduction

The aflatoxin is one of the most important factors in the poultry industry and also is a secondary product of the metabolism of many species of fungi such as *Aspergillus flavus*, with many types (B1, B2, G1, and G2) [1]. The risk of contamination diets with aflatoxin caused poor body performance, immunosuppression by the effect on immune organs, increased susceptibility to other diseases, and increased mortality [2].

Liver represents the main target organ in aflatoxicosis that will cause several biochemical changes through impairing or disturbance in the metabolism of lipids, vitamins, proteins, nucleic acids, amino acids, and liver enzymes [3].

The levels of liver enzymes mainly alanine transaminase (ALT) and aspartate transaminase (AST) enzymes increase during aflatoxicosis in chicken and turkey and represent the severity and liver damage due to infection [4,5]. There are many of ongoing studies at present to reduce or antagonize their harmful effects by commercial products due to high levels of toxicity even at low doses.

Levamisole is widely used in poultry farms as an immunostimulatory agent and minimize the bad effect of aflatoxin. The synthetic anthelmintic drug for many species of animals has many therapeutic and immunological functions such as immune stimulants in modulation activity leukocyte toxicity, respiratory problems, antibody response, and macrophage activating factor [6], as well as impacts to improve humoral and cell-mediated immunity [7]. The Vitamin E and selenium considered to be an essential trace nutrient for animals and humans. The supplementation of Vitamin E and selenium in poultry diets is one of the important components of the antioxidant defense system. They help to protect cell membranes from peroxidative damage [8]. Furthermore, it has been suggested that there is a synergistic relationship between Vitamin E and selenium [9].

An attempt has been made in this study to investigate the effect of levamisole and Vitamin E and selenium to boost immunity and enhance the level of protection against the harmful effect of aflatoxin.

## Materials and Methods

### Ethical approval

The study was approved for research purpose by the Ethics Committee at College of the Veterinary Medicine, University of Baghdad-Iraq (2016).

### Experimental design

The experiment included (200) 1-day-old broilers Ross 308 (Turkey source) mixed sexes. They were divided into four equal (4) groups, 50 chicks for each group (5 m per cage). The experimental period was extended to 35 days with full hygiene conditions and vaccination programs and under complete control of temperature and humidity. The feed was given to all groups *(ad libitum)* in all the experiment (35 days) which contain aflatoxin (B1) at the dose (0.8 ppm). The experiment was conducted at the poultry farm/University of Baghdad - College of Veterinary Medicine. Experimental groups are divided as follows:

T1:Commercial product of levamisole (Uevmisole^®^) 1 ml/contain levamisole hydrochloride 118/mg. At dose (0.2 ml/kg - body weight) with drinking water.
T2:Treated with (0.5 ml/L) of commercial products UVEDCO ES^®^ Vitamin E 80 mg selenium 1.6 mg provided with drinking water.
T3:Fed aflatoxin (B1) in dose (0.8ppm) (control positive).
T4:Control negative (uncontaminated diet).

All chickens in groups except (T4) were given a diet containing aflatoxin from 1 day old to 35-day olds. All additives (levamisole, Vitamin E, and selenium) were given through drinking water for all days of the experiment.

### Sample collection

Five blood samples were collected randomly from each group at 35 days old for measuring the concentrations of the AST enzyme and ALT enzyme. The blood samples were centrifuged at 1500×RPM for 15 min, then serum was harvested and stored at –20°C until analyzed by Automatic Biochemical Analyzer system-KENZA 240TX. The automatic method was connected to the computer to record the results. Specimens were taken from the organs of a bird (thymus and Harderian gland) at 25 and 35 days to show the histopathological changes.

### Feed samples detection

The diet belongs to the fodder shipment which was excluded according to the results of the Iraqi qualitative control while the diet of the control negative was free of aflatoxin. All groups were given the dit *ad libitum* for 35 days. The concentration of aflatoxin (B1) was 0.8ppm, which was measured by ELISA and HPLC methods [10].

### Feed contents

The feed contents were used one type of diet on the all periods of the experiment as shown in Table-1.

**Table-1:** Ingredients and composition of the basal diet of broiler.

Ingredients	Kg/Ton	Percentage
Corn	550 kg	55
Wheat	207 kg	20.7
Soybean meal	215 kg	21.5
Calcium	10 kg	1.0
Premix 1%	15 kg	1.5
Salt	3 kg	0.3
Energy %	3170	
Protein %	19.6	

### Histopathology

The histopathological specimens of all processing and staining (hematoxylin and eosin stain) were made under the routine procedure to study the histopathological changes [11].

### Statistical analysis

The Statistical Analysis System (2012) program was used to analyze the data. One-way analysis of variance was performed. Least significant difference test was used to assess the significant differences among means. p<0.05 was considered statistically significant [12].

## Results

The result of liver enzymes (ALT and AST) at 35 days old showed that the level of ALT and AST was significantly (p<0.05) higher than the negative control. However, the increase in the ALT (23.76 IU/L) and AST (264.00 UI/L) in the Vitamin E+selenium group was lower than other groups (Table-2).

**Table-2:** Effect group in liver enzymes (ALT and AST) levels.

Groups at (35 days old)	ALT (IU/L)	AST (IU/L)
T1/levamisole	29.66±1.94^B^	282.0±9.52^AB^
T2/vitamin E and SE	23.76±1.36^C^	264.0±7.91^B^
T3/cont. +ve	36.46±2.07^A^	306.4±11.43^A^
T4/cont. -ve	7.80±0.62^D^	145.8±6.27^C^
LSD value	5.483*	24.614*

* (p<0.05).

Means with a different letter in the same column significantly different (p<0.05). LSD=Least significant difference, AST=Aspartate transaminase, ALT=Alanine transaminase

**Figure-1:**
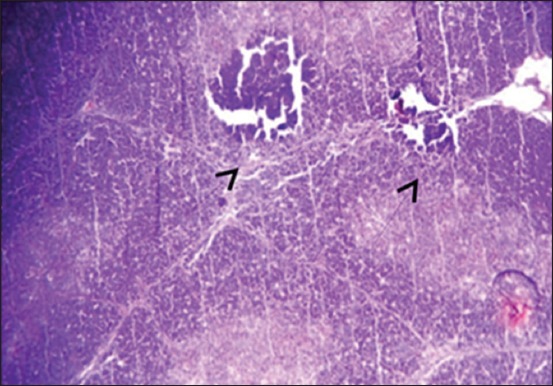
Histological section in Thymus 1^st^ group at (25 day old): There is multifocal areas of necrosis (arrow) (H and E, 10×).

**Figure-2:**
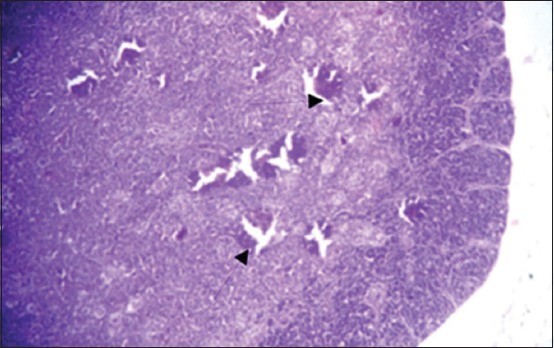
Histological section in Thymus 2^nd^ group at (25 day old): Multifocal area of necrosis (arrow) (H and E, 20×).

**Figure-3:**
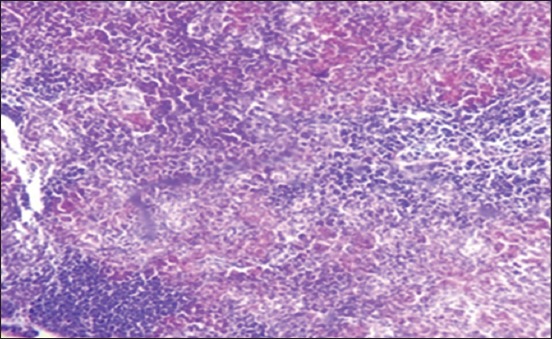
Histological section in Thymus 3^rd^ group at (25 day old): Depletion of the thymus lobules and necrosis (arrow) (H and E, 20×).

The results of histopathological changes occurred in thymus at 25 days old, showed that the lesion, in 1^st^ group, there are multifocal areas of necrosis in the cortex of thymus globules (Figure-1). The 2^nd^ group showed moderate multifocal areas of necrosis (Figure-2). The 3^rd^ group showed increased clarity of depletion in the thymus lobules and necrosis (Figure-3). Concerning the 4^th^ group, it was still normal.

**Figure-4:**
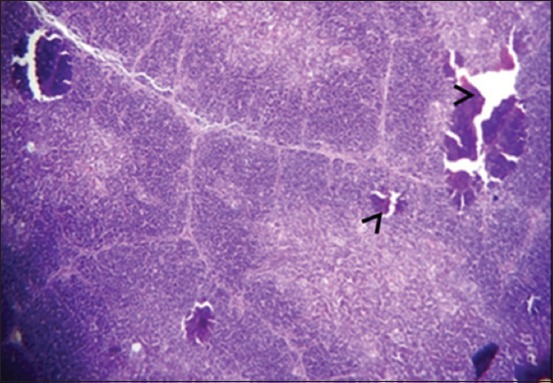
Histological section in Thymus 1^st^ group at (35 day old): There is multifocal areas of necrosis (arrow) (H and E, 20×).

**Figure-5:**
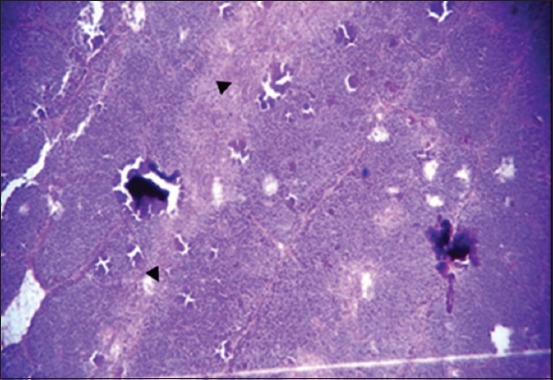
Histological section in Thymus 2^nd^ group at (35 day old): Multifocal necrosis in cortex and medulla of thymes lobules (arrow) (H and E).

**Figure-6:**
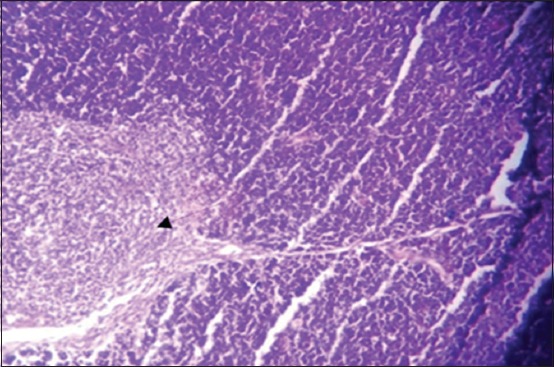
Histological section in Thymus 3^rd^ group at (35 day old): Focal sub capsular granuloma (arrow) (H and E, 20×).

**Figure-7:**
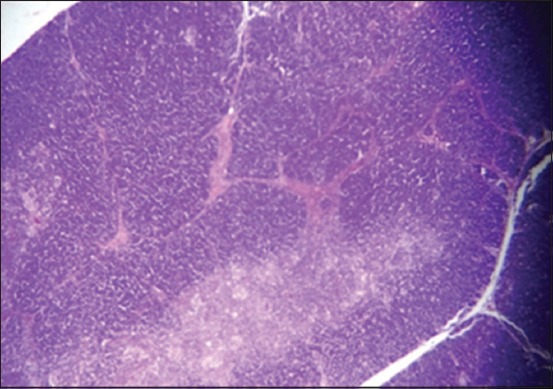
Histological section in Thymus 4^th^ group in all ages normal thymus gland (H and E, 10×).

Moreover, at 35 days old, in the 1^st^ group, the necrosis becomes more severe and there are multifocal areas of necrosis in the gland parenchyma (Figure-4). In the 2^nd^ group, results showed multiple areas of focal necrosis in the cortex and medulla of thymus lobules (Figure-5). The necrotic area appears as empty space, while other areas show calcification. For the 3^rd^ group, the thymus showed focal subcapsular granuloma consisting of macrophages and epithelioid cells (Figure-6). The 4^th^ group was normal (Figure-7).

**Figure-8:**
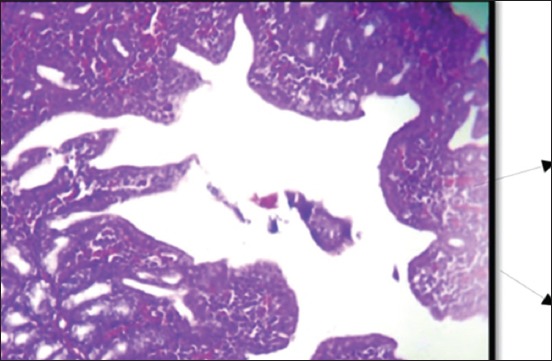
Histological section Harderian gland 1^st^ group at (25 day old) congestion of blood vessels (H and E, 10×).

**Figure-9:**
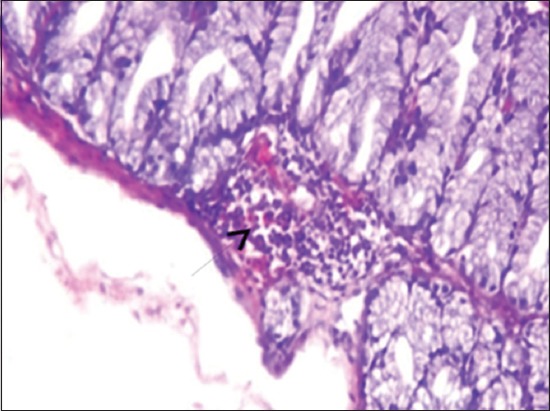
Histological section Harderian gland 2^nd^ group at (25 day old) congestion of blood vessels and focal aggregation of MNCs (arrow) (H and E, 20×).

**Figure-10:**
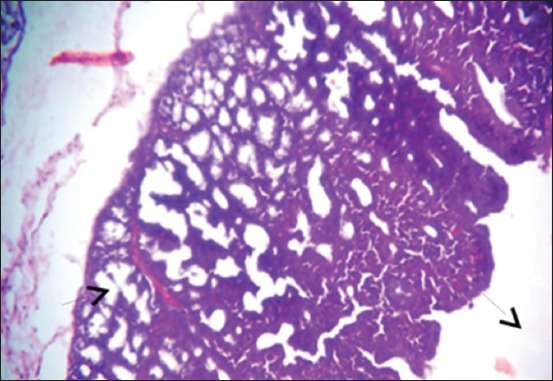
Histological section Harderian gland group 3^rd^ at (25 day old) multiple areas of necrosis (arrow) (H and E, 20×).

In Harderian gland, the changes at 25-day-old lesion were shown in the 1^st^ group. There is a congestion of blood vessels (Figure-8), with few areas of necrosis. The 2^nd^ group showed a congestion of blood vessel, mild hyperplasia of the glandular epithelia, and focal aggregation of MNCs in the interstitial tissue, especially in the subcapsular area (Figure-9), while the 3^rd^ group showed multiple areas of necrosis (Figure-10).

**Figure-11:**
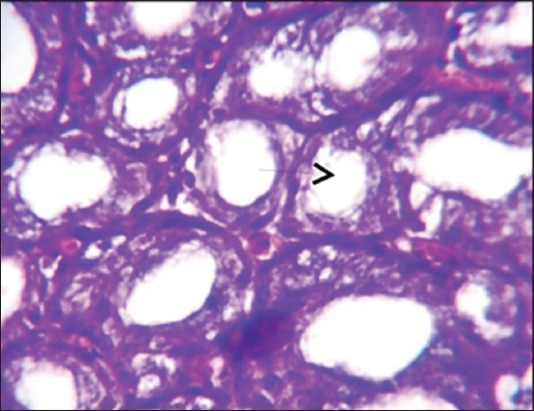
Histological section Harderian gland 1^st^ group at (35 day old (normal structure of Harderian gland (H and E, 40×).

**Figure-12:**
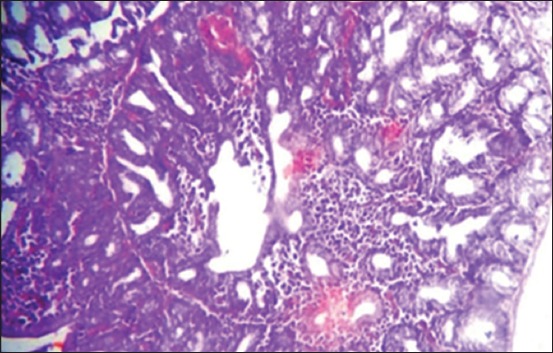
Histological section Harderian gland 2^nd^ group at (35 day old) congestion of blood vessels, hemorrhage (arrow) and MNCs infiltration in the interstitial tissue (H and E, 20 ×).

**Figure-13:**
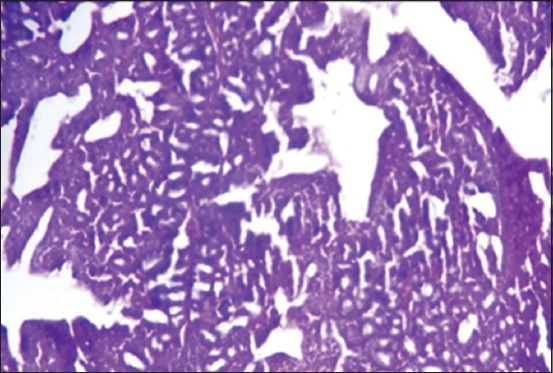
Histological section Harderian gland 3^rd^ group at (35 day old (severe necrosis (arrow) in the epithelia of alveoli of Hadrian (H and E, 20×).

**Figure-14:**
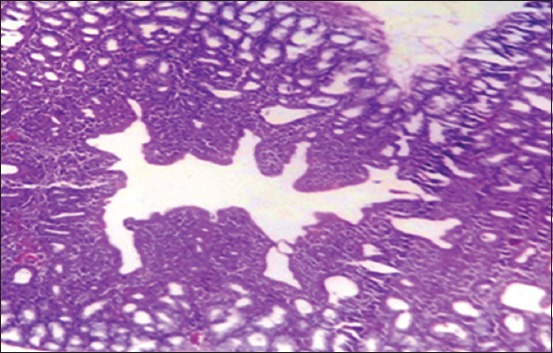
Histological section Harderian gland- 4^th^ group in all ages normal gland (H&E Stain- 10 ×).

At 35 days old, the Harderian gland in the 1^st^ group showed normal architecture (Figure-11). The 2^nd^ group showed infiltration of MNCs in the interstitial tissue (Figure-12), congestion of blood vessels, and hyperplasia of glandular epithelia. The lesion in the 3^rd^ group was characterized by severe diffuse necrosis (Figure-13). The Harderian gland at the 4^th^ group showed normal histological section at the end of the experiment (Figure-14).

## Discussion

In general, the levels of liver enzymes increased when compared with the normal value in T4. Results showed that the best protective effect on the liver enzymes (ALT and AST) was in the Vitamin E and selenium (T2) followed by the levamisole (T1). These results agreed with the results obtained by Saad [13] who studied the effect of different levels of feed additives on liver enzymes (ALT and AST) and demonstrated the limited impact of levamisole when compared with different levels of Digestarom. Similar results were found in the pigs [14].

The Vitamin E and selenium represent a good antioxidant agent. When they add at the dose 250 mg Vitamin E/kg diet, they improve and increase the level of glutathione decreasing peroxidase secretion in the liver and led to reducing the liver damage along with the levels of liver enzymes to the normal range [15]. Whereas, the effect of Vitamin E and selenium on liver damage caused by cyclophosphamide in rat was determined in liver enzymes (ALT and AST). The result revealed a significant increase in liver enzyme activity in all treated groups with cyclophosphamide compared to the control group and led to damage to liver tissue due to cyclophosphamide [16].

The histopathological results indicated the best additives to improve and reduce the harmful effect of aflatoxin was vitamin E and selenium in the 2^nd^ group then followed levamisole (1^st^ group), respectively, as compared with the positive control in the 3^rd^ group, while the negative control (4^th^ group) remained normally in different organs and ages.

The result of histopathological changes in this study agreed with other previous studies, concerning the role of levamisole in enhancing the immune response. The levamisole decreased depletion of lymphocyte and increased proliferation of lymphocyte [17]. Furthermore, other studies revealed that the levamisole effect was on cecal tonsils, bursa, and less profoundly on thymus in normal broilers [18]. Moreover, administered levamisole as adjuvants in vaccine showed no histopathological changes in any organs in fish [19]. The Vitamin E and selenium reduced the effects of aflatoxin on the histopathological changes. Similar results were obtained by Ali [17] who reported that Vitamin E and selenium additives led to improve the immune response and lymphoid organs by modulating the metabolic end product or by activation the glutathione peroxidase. The aflatoxin is inactivated by binding with glutathione-s-transferase and excreted through urine and bile. Moreover, the Vitamin E and selenium prevent a gradual degeneration of the epithelium and an accompanying depletion of lymphocytes in lymphoid organs also reducing impaired of thymus growth [20,21].

## Conclusion

This study confirmed the harmful effect of aflatoxin besides its difficult removal or reducing its effect. The study concluded the protective effect of Vitamin E and levamisole by reducing harmful impacts of aflatoxin through the antioxidant effect of Vitamin E and levamisole and by improving the liver enzymes and histopathological changes due to the toxin. Hence, it is very imperative to protect the diet from aflatoxin contamination.

## Authors’ Contributions

Amjed H. Ulaiwi is the sole author.

## References

[1] Gokhan E, Din E, Mehmet A, Fatma S, Levent A (2005). The effects of aflatoxin and sodium bentonite combined and alone on some blood electrolyte levels in broiler chickens. Turk. J. Vet. Anim. Sci.

[2] Sefa E, Zeynep E, Suat E, Ramazan B.A.L (2005). Efficacy of tribasic copper chloride (TBCC) to reduce the harmful effects of aflatoxin in broilers. Turk. J. Vet. Anim. Sci.

[3] Ellis W.O, Smith J.P, Simpson B.K (1991). Aflatoxin in food occurrence, biosynthesis, effects on organisms, detection and methods of control. Crit. Rev. Food Sci. Nutr.

[4] Cheng Y.H, Shen T.F, Pang V.F, Chen B.J (2000). Effects of aflatoxin and carotenoids on growth performance and immune response in mule ducklings. Comp. Biochem. Physiol.

[5] Quist C.F, Bounous D.I, Kilburn J.V, Nettles V.F, Wyatt R.D (2000). The effect of dietary aflatoxin on wild turkey poults. J. Wildl. Dis.

[6] Habibi M, Mohammad A.Z, Hasan G, Reza S, Farid Y (2012). Effects of levamisole on the immune response of broilers against Newcastle disease vaccines. Afr. J. Pharm. Pharmacol.

[7] Sanda M.E, Anene B.M, Owoade A (2008). The effect of levamisole as an immunomodulator in cockerels vaccinated with a Newcastle disease vaccine. Int. J. Poult. Sci.

[8] Ibrahim M.T, Eljack B.H, Fadlalla I.M (2011). Selenium supplementation of broiler diets. Anim. Sci. J.

[9] Nemat Z, Nasroallah M.K, Ebrahim E (2013). The effects of different levels of vitamin-E and organicselenium on performance and immune response oflaying hens. Afr. J. Biotechnol.

[10] Akbar P, Seyyed N, Mohammad H, Mohammad A, Mehdi B (2011). Comparison of HPLC and ELISA for determination of aflatoxin concentration in the milk and feeds of dairy cattle. J. Res. Agri. Sci.

[11] Luna L.G (1968). Manual of Histologic Staining Methods of the Armed Force Institute of Pathology.

[12] SAS (2012). Statistical Analysis System, User's guide. Statistical. Version 9.

[13] Saad M.H (2016). Effect of Adding Different Concentrations of Digestarom and Levamisole on some Immune and Productive Aspects in Broiler. (Thesis). Degree of Master of Science in Veterinary Medicine-Poultry Diseases. College of Veterinary Medicine, University of Baghdad-Iraq.

[14] Hrvoje V, Renata B.R, Vladimir M, Frane B, Zeljko G, Marko S, Ivan F, Drazen D, Damjan G, Ivica V (2016). The influences of immune modulation with levamisole and polyoxyethylene-polyoxypropylene copolymers on the immunohematological, serum biochemical parameters and intestinal histocytomorphology of weaned pigs. Vet. Arhi.

[15] Samar S.T, Kamel M.H, Ibrahim M.I (2014). The effect of dietary supplementation of some antioxidants on performance, oxidative stress, and blood parameters in broilers under natural Summer conditions. J. World's Poult. Res.

[16] Mozhdeh K, Mahmood N (2014). Studying the effect of vitamin E and selenium on liver enzymes in chemotherapy rat with cyclophosphamide. Biosci. Biotech. Res. Asia.

[17] Ali E.J (2014). Comparative study between additives on immune response of infectious bursal disease vaccine in broiler feed diet. Int. J. Sci. Natu.

[18] Hamed S, Shomal T, Akbarian R, Zandi A.S, Sdaeghi S (2016). Effect of levamisole on active antibody titer and histomorphometric parameters of immune organs in broiler chickens. Bulg. J. Vet. Med.

[19] Morrison R.N, Nowak B.F, Carson J (2001). The histopathological effects of a levamisole-adjuvanted Vibrio anguillarum vaccine on atlantic salmon*(Salmo salar*L**.). Aquaculture.

[20] Marsh J.A, s G.F, Whitacre M.E, Dietert R (1986). Effect of Selenium and Vitamin E Dietary Deficiencies on Chick Lymphoid Organ Development. Proceeding of the Society for Experimental Biology and Medicine.

[21] Amjed H. Ulaiwi (2017). Comparative studies between probiotic and vitamin E and selenium to reduce the effect of aflatoxin in broiler chicken. J. Entomol. Zool. Stud.

